# Demography and selection analysis of the incipient adaptive radiation of a Hawaiian woody species

**DOI:** 10.1371/journal.pgen.1009987

**Published:** 2022-01-21

**Authors:** Ayako Izuno, Yusuke Onoda, Gaku Amada, Keito Kobayashi, Mana Mukai, Yuji Isagi, Kentaro K. Shimizu

**Affiliations:** 1 Department of Forest Molecular Genetics and Biotechnology, Forestry and Forest Products Research Institute, Tsukuba, Japan; 2 Department of Evolutionary Biology and Environmental Studies, University of Zurich, Zurich, Switzerland; 3 Graduate School of Agriculture, Kyoto University, Kyoto, Japan; 4 Kihara Institute for Biological Research, Yokohama City University, Yokohama, Japan; University of Cologne, GERMANY

## Abstract

Ecological divergence in a species provides a valuable opportunity to study the early stages of speciation. We focused on *Metrosideros polymorpha*, a unique example of the incipient radiation of woody species, to examine how an ecological divergence continues in the face of gene flow. We analyzed the whole genomes of 70 plants collected throughout the island of Hawaii, which is the youngest island with the highest altitude in the archipelago and encompasses a wide range of environments. The continuous *M*. *polymorpha* forest stands on the island of Hawaii were differentiated into three genetic clusters, each of which grows in a distinctive environment and includes substantial genetic and phenotypic diversity. The three genetic clusters showed signatures of selection in genomic regions encompassing genes relevant to environmental adaptations, including genes associated with light utilization, oxidative stress, and leaf senescence, which are likely associated with the ecological differentiation of the species. Our demographic modeling suggested that the glaberrima cluster in wet environments maintained a relatively large population size and two clusters split: polymorpha in the subalpine zone and incana in dry and hot conditions. This ecological divergence possibly began before the species colonized the island of Hawaii. Interestingly, the three clusters recovered genetic connectivity coincidentally with a recent population bottleneck, in line with the weak reproductive isolation observed in the species. This study highlights that the degree of genetic differentiation between ecologically-diverged populations can vary depending on the strength of natural selection in the very early phases of speciation.

## Author summary

Knowledge about how genetic barriers are formed between populations in distinct environments is valuable to understand the processes of speciation and conserve biodiversity. *Metrosideros polymorpha*, an endemic woody species in the Hawaiian Islands, is a good system to study developing genetic barriers in a species, because it colonized the diverse environments and diversified the morphology for a relatively short period of time. We analyzed the genomes of 70 *M*. *polymorpha* plants from a broad range of environments on the island of Hawaii to infer the current and past genetic barriers among them. Currently, *M*. *polymorpha* plants growing in different environments have substantially different genomes, especially at the genomic regions with genes putatively controlling physiology to fit in distinct environment. However, in its history, they had hybridized with one another, possibly because plants formerly growing in different environments came into close contact due to the climate changes. It is suggested that genetic barriers can easily strengthen or weaken depending on environments splitting the ecology of a species before reproductive isolation becomes complete.

## Introduction

Adaptation to distinct resources or environments often causes genetic differentiation in a species [1]. The genetic differentiation can increase and result in speciation, particularly when an ecological divergence is allopatric [2] and/or adaptive traits are associated with non-random mating [3]. However, ecologically-divergent populations do not always develop reproductive isolation in nature [4] and we have still limited knowledge about how an ecological divergence continues in the face of gene flow.

*Metrosideros polymorpha* Gaud. (Myrtaceae), which is an endemic and dominant species in the native forests in the Hawaiian Islands, represents a unique example of incipient adaptive radiation of a woody species. After colonizing the Hawaiian Islands 3.1–3.9 million years ago [5,6], the species has adapted to a wide range of environments: it grows from sea level to the alpine tree line and from dry substrates in early successional stages to wet and mature forest soils [7,8]. The morphology of the leaves and other organs of the plant and its physiology are diverse and vary with the environments of different habitats [7,9–12]. Morphological variation in a common garden suggests that most of the phenotypic differences are genetically determined [10,12,13]. Home site advantage shown in reciprocal transplantation experiments indicates that phenotypic differentiation is associated with fitness [14]. Despite efforts in recent genomic studies [15,16], the genetic regions and selective forces associated with phenotypic differences are still unclear.

The genetic differentiation between ecologically-divergent populations in *M*. *polymorpha* is not evident. *Metrosideros polymorpha* plants are classified into different varieties based on morphological characteristics and habitat environments [17] and genetic differentiation has been found for some varieties [18]. However, in the field, continuous forest stands of *M*. *polymorpha* are comprised of not only plants with representative morphologies of particular varieties but also plants with intermediate morphologies. This suggests that hybridization between varieties is common in the species and reproductive isolation between varieties is likely to be incomplete [19]. Moreover, phylogenetic and population genetic studies focusing on *Metrosideros* species/varieties across islands showed the genetic distances between the same varieties in different islands is larger than those between different varieties within islands despite its phenotypic similarity, suggesting that the radiation occurred repeatedly in each island [5,20]. Therefore, varieties across islands may not represent evolutionary units that account for the spatiotemporal scale of the ecological divergence in *M*. *polymorpha* or more complex demographic history should be considered.

This study aimed to establish how *M*. *polymorpha* maintained the ecological divergence on the island of Hawaii. As the island of Hawaii is the largest, youngest island with highest altitude in the archipelago, it encompasses the most variety of climates associated with the variation in altitude and substrate age compared to other islands [21]. Therefore, the *M*. *polymorpha* populations on the island is a suitable system to study the most recent and extensive adaptive evolution of the species and gain insight into demographic and selective forces sustaining an ecological divergence. Previously, population genomic analyses on the island of Hawaii focused on nine populations along an altitudinal transect [15] and the riparian adaptation of a rare variant var. *newelii* [16]. With the aid of a genome assembly composed of chromosome-scale pseudo-molecules [22], we investigated the whole-genome polymorphisms of 70 samples from 55 localities covering the altitudinal range, lava types, and five varieties from across the island. Firstly, we examined the population structure of *M*. *polymorpha* on the island and identified the primary evolutionary units that are genetically differentiated along environmental gradients across the island. Secondly, we employed multiple methodologies for selection scan and identified candidate genes with relevant functions for environmental adaptation. Lastly, we used coalescent modeling to infer the demographic history of *M*. *polymorpha*. We discuss how ecological divergence has been maintained in this species despite the presence of gene flow.

## Results

**Fig 1:**
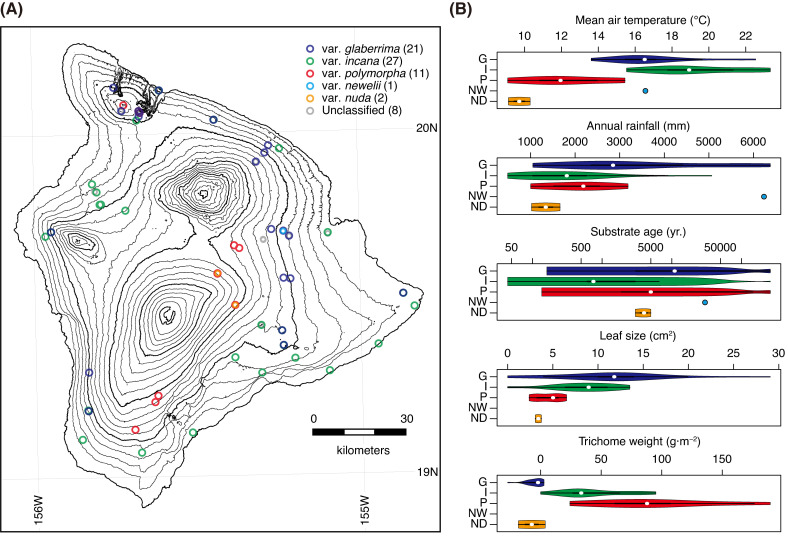
**(A)** Sample localities. Colors indicate varieties classified based on leaf morphology: var. *glabrrrima* (blue), var. *incana* (green), var. *polymorpha* (red), var. *newelii* (cyan), and var. *nuda* (orange). Unclassified samples with intermediate morphologies are indicated with gray. Numbers in parenthesis indicate sample sizes. **(B)** The variation in the mean air temperature, annual rainfall, substrate age, leaf size, and trichome weight of five *M*. *polymorpha* varieties. Unclassified samples are not included. G, I, P, NW, and ND indicate var. *glabrrrima*, var. *incana*, var. *polymorpha*, var. *newelii*, and var. *nuda*, respectively. Leaf size and trichome weight were not measured for var. *newelii*.

We resequenced the whole genomes of 70 *M*. *polymorpha* plants from 55 sites on the island of Hawaii with a 29.6-fold mean sequencing coverage (Fig 1A). The 55 sites covered the environmental range of the habitats on the island, spanning 15–2,373 m in altitude, 9°C –23°C in mean annual air temperature, 486–6,379 mm in annual precipitation, and from <100 to over 250,000 years old in substrate age. The 70 samples included the five varieties known to occur on the island of Hawaii: var. *glaberrima*, var. *incana*, var. *polymorpha*, var. *nuda*, and var. *newelii*, and unclassified samples with intermediate characteristics in morphology (Fig 1A). The former three varieties are common on the island. Var. *glaberrima* has large, glabrous leaves and mainly grows in wet forests [17]. Var. *polymorpha* has small and extremely pubescent leaves and grows in subalpine zones [17]. Var. *incana* has mid-sized leaves with short trichomes on the surface and grows on young and dry substrates in the lowlands [17]. The remaining two varieties are rare, so fewer samples were included for them: Var. *nuda* shares habitats with var. *polymorpha*, but it has tiny glabrous leaves, and Var. *newelii* only grows along a river and has riparian-formed narrow leaves [23]. Our quantitative measurements of leaf size and the weight of trichomes revealed that leaf traits were remarkably different, even within varieties (Fig 1B and S1 Table).

### Population structure

**Fig 2:**
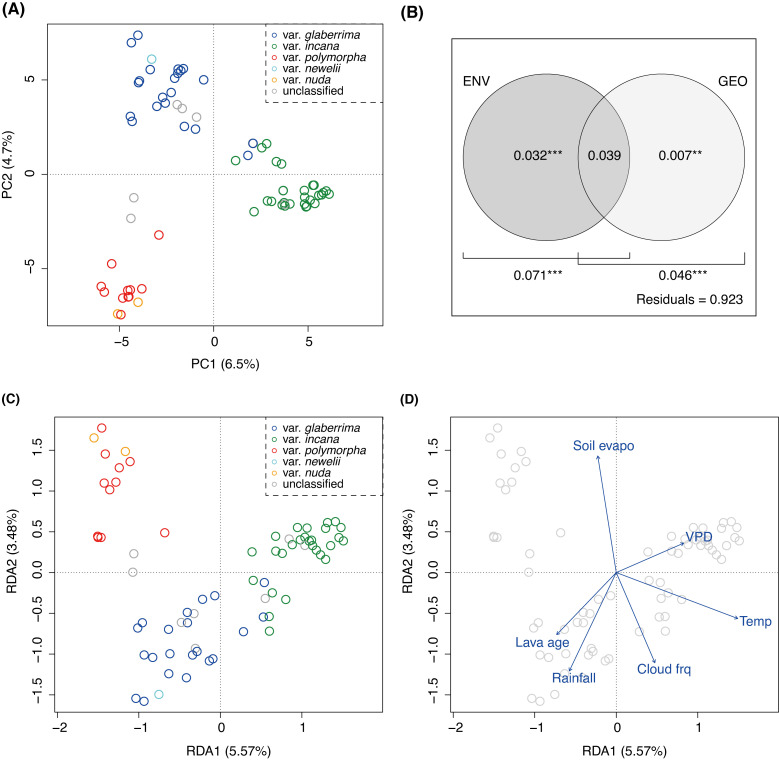
Population structure in *Metrosideros polymorpha* on the island of Hawaii. **(A)** Scatter plot of principal components (PC) 1 and 2. Colors indicate varieties classified based on leaf morphology: var. *glabrrrima* (blue), var. *incana* (green), var. *polymorpha* (red), var. *newelii* (cyan), and var. *nuda* (orange). **(B)** Proportion of genetic variation explained by geographic (GEO) and environmental (ENV) distances. Statistical significance of each variable was examined by a permutation test (***: *p* < 0.001; **: *p* < 0.05) **(C)** Scatter plot of the first two components of redundancy analysis (RDA). Colors indicates as (a). **(D)** Environmental variables fitted on the RDA plot. The environmental variables included substrate age (Lava age), mean air temperature (Temp), annual precipitation (Rainfall), cloud frequency (Cloud frq), soil evaporation (Soil Evapo), and vapor pressure deficit (VPD).

Population structure was investigated based on the genotypes at the 16,972 four-fold degenerate single nucleotide polymorphisms (SNPs). In the principal component analysis (PCA), the PC1 and PC2 scores showed that the 70 plants were differentiated into three groups, which mostly corresponded to the three common varieties found on the island of Hawaii: var. *glaberrima*, var. *incana*, and var. *polymorpha* (Fig 2A). Var. *newelii* and var. *nuda* belonged to the var. *glaberrima* and var. *polymorpha* clusters, respectively, despite the clear differences in morphology. The population clustering assuming two or three ancestries in the ADMIXTURE [24] supported genetic differentiation among the three common varieties (S1 Fig), in contrast to larger cross-validation errors for the increased number of ancestries (*K*) (S2 Fig). Further genetic differentiation was shown in var. *glaberrima* at *K* = 4 and in var. *incana* at *K* = 5 (S1 Fig).

To evaluate the effect of geography and environment on the genetic differentiation, we quantified the proportion of genetic variation explained by geographic and environmental distances using redundancy analysis (RDA). As a result, both geographic and environmental distances significantly accounted for the genetic variation of the 70 *M*. *polymorpha* plants. Environmental distances accounted for 7.1% (*p* < 0.001) of the genetic variation and 3.2% (*p* < 0.001) while controlling for geographic distances (Fig 2B). The proportion of genetic variation explained by geographic distances was 4.6% (*p* < 0.001), but after controlling the confounded fractions, the proportion decreased into 0.7% (*p* < 0.05; Fig 2B). Therefore, the genetic variation among the 70 plants was more affected by environmental differences than by geographic distances.

Scatter plot of the first two components of RDA (RDA1 and 2) suggested that the three primary genetic clusters identified in PCA and ADMIXTURE were differentiated according to the environment of habitat (Fig 2C and 2D). The cluster of var. *polymorpha* and var. *nuda* was primarily distributed in subalpine zones, where cloud does not occur frequently (Cloud frq in Fig 2D). The low mean air temperature (ca. 10°C; Temp in Fig 2D) also makes the environment of the habitat harsh. The habitat of var. *incana* was characterized by high mean air temperature and high vapor pressure deficit (VPD in Fig 2D), indicating that the genetic cluster is exposed to dry and hot conditions. The cluster of var. *glaberrima* and var. *newelii* occurred in wet environments in mature forests on old substrate (Lava age in Fig 2D), in which the amount of precipitation exceeded 6,000 mm per year, and the vapor pressure deficit was less than 300 Pa (Rainfall and VPD in Fig 2D).

In sum, *M*. *polymorpha* populations were mainly differentiated according to environmental differences on the island of Hawaii. Hereafter, we focus on the three genetic clusters according to the plot of their PC1 and 2 scores (Fig 2A), RDA1 and 2 scores (Fig 2C), and the population clustering at *K* = 3 (S1 Fig), and we refer to them as the glaberrima (including var. *glaberrima* and var. *newelii*), incana (including var. *incana*), and polymorpha (including var. *polymorpha* and var. *nuda*) genetic clusters.

### Genetic diversity

**Fig 3:**
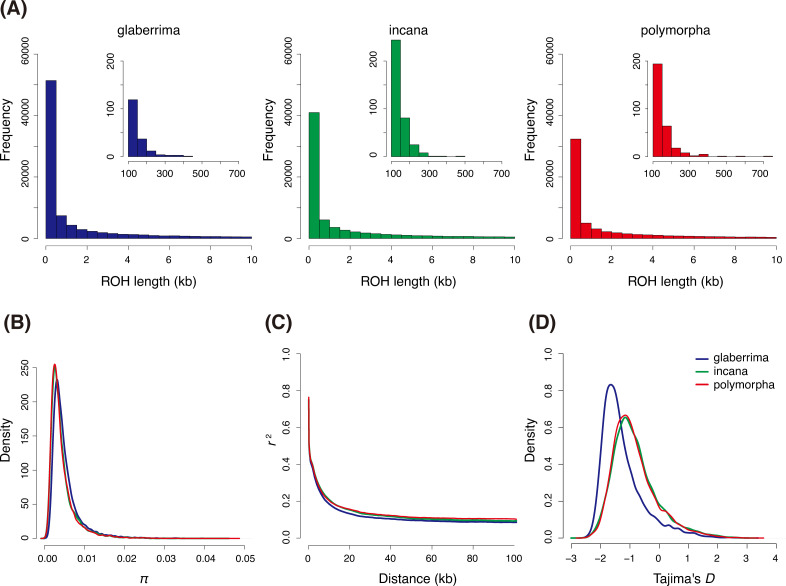
Genetic diversity in the three primary *Metrosideros polymorpha* genetic clusters on the island of Hawaii. **(A)** The distribution of runs of homozygosity (ROH) with lengths less than 10 kb (main panels) and more than 100 kb (inset panels). **(B)** Probability distribution of the nucleotide diversity (*π*). **(C)** Decay of linkage disequilibrium (*r*^2^) with physical distance. **(D)** Probability distribution of Tajima’s *D*.

The three *M*. *polymorpha* clusters showed different patterns of genetic polymorphisms. In the glaberrima cluster, only 0.2% of runs of homozygosity (ROH) exceeded 100 kb (Fig 3A). In contrast, in the incana and polymorpha clusters, the distribution of ROH shifted to longer ranges (Fig 3A). In the polymorpha cluster, 0.47% of ROHs developed more than 100 kb up to 723 kb (Fig 3A). Slightly larger polymorphism (*π*: 0.00522 ± 0.00367) was observed in the glaberrima cluster than the other two genetic clusters (*π*: 0.00453 ± 0.00353 and 0.00445 ± 0.0035 in the incana and polymorpha cluster, respectively; Fig 3B). The linkage disequilibrium (LD) decays dropped to *r*^2^ = 0.2 within 10 kb in all the genetic clusters, although the tendency was the most prominent in the glaberrima cluster (Fig 3C). The negatively skewed distributions of Tajima’s *D* indicated that all the genetic clusters, particularly the glaberrima cluster, have rare variants and experienced recent population expansions (Fig 3D).

### Genetic regions under selection

**Fig 4:**
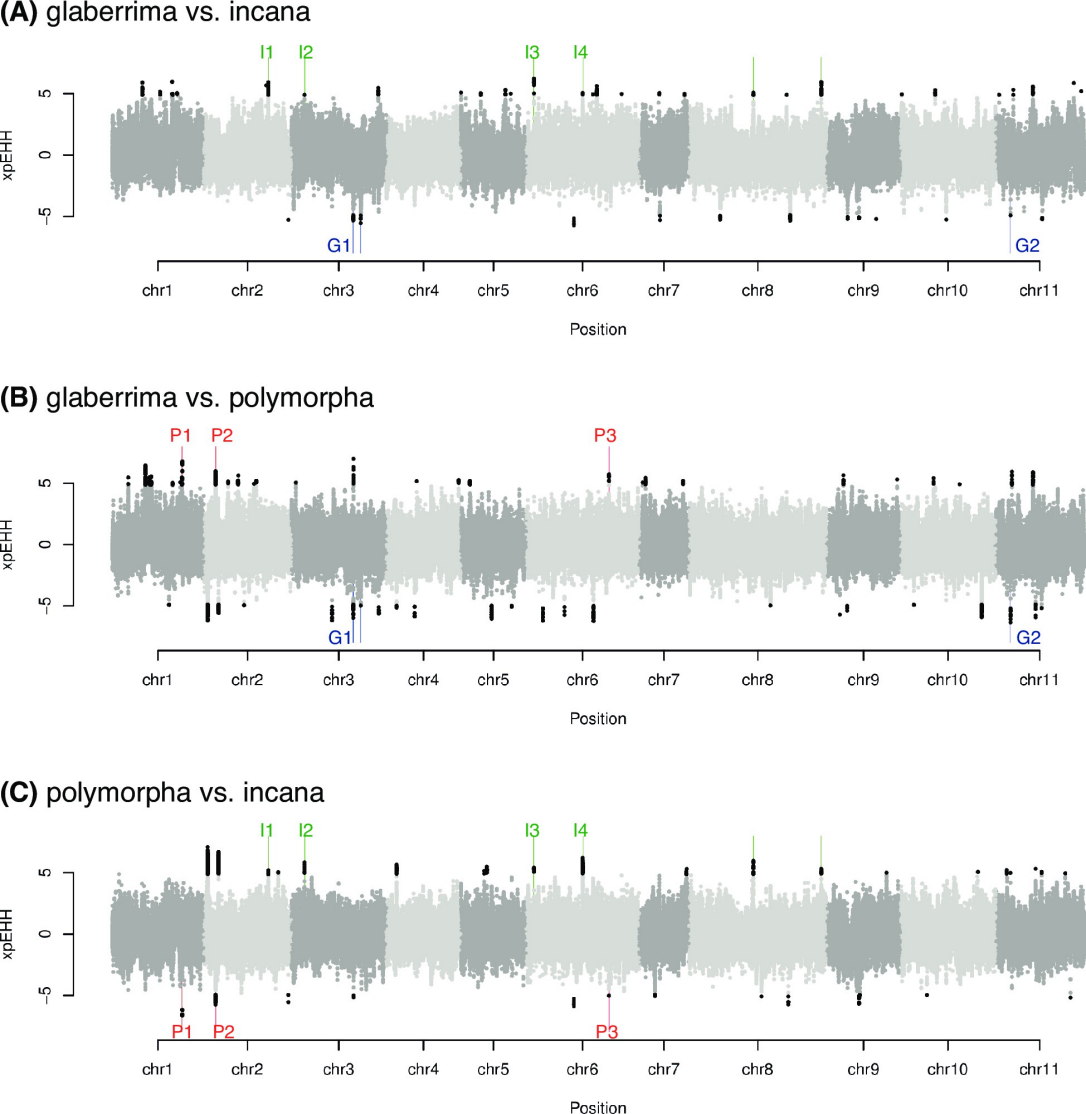
Cross-population extended haplotype homozygosity (xp-EHH) scores calculated between **(A)** the glaberrima and incana clusters, **(B)** the glaberrima and polymorpha clusters, and **(C)** the polymorpha and incana genetic clusters. The genomic regions with significantly extended haplotype homozygosity (EHH) against the other genetic clusters are indicated by black dots. The three, six, and three regions in which the glaberrima, incana, and polymorpha clusters showed significantly EHH against both of the other genetic clusters were indicated by blue, green, and red vertical lines, respectively. G1–G2, I1–I4, and P1–P3 indicated the EHH regions that overlapped with significant regions detected in at least one of the other methodologies for selection scan (i.e., sweep statistics, *F*_ST_, *D*_XY_, *π*, and Tajima’s *D*).

To search for candidate genomic regions under selection, we here employed approaches using extended haplotype homozygosity, sweep scan and *F*_ST_ combined with population genetic statistics such as Tajima’s *D*, nucleotide diversity (*π*), and absolute genetic distance (*D*_XY_). As statistical power to detect selection varies among methodologies [25], combining results from multiple methodologies can help to identify candidate genomic regions with prominent signature of selection. First, we compared haplotype homozygosity between each pair of the three genetic clusters. The glaberrima, incana, and polymorpha clusters respectively showed extended haplotype homozygosity (EHH) at 33, 45, and 38 regions compared with at least one other genetic cluster and at 3, 6, and 3 regions compared with both of two other genetic clusters (Fig 4).

Second, the signatures of selective sweep were examined using RAiSD [26]. RAiSD detected the signatures at 136, 89, and 60 regions in the glaberrima, incana, and polymorpha cluster, respectively (S3 Fig). Third, local deviations of genetic differentiation were tested based on *F*_ST_ calculated for non-overlapping 20 kb windows. Genome-wide *F*_ST_ was 0.103 ± 0.091, 0.099 ± 0.09, 0.132 ± 0.116 between the glaberrima and incana, the glaberrima and polymorpha, and the incana and polymorpha cluster, respectively. We identified outlier *F*_ST_ at 17, 11, and 40 windows between the glaberrima and incana, the glaberrima and polymorpha, and the incana and polymorpha clusters, respectively (S4 Fig). Finally, we computed Tajima’s *D*, π, and *D*_XY_ for non-overlapping 20 kb windows and identified outlier windows. By combining the results from these analyses, we identified a total of nine EHH regions with evidence from other methodologies (Table 1).

**Table 1:** Genomic regions with significantly extended haplotype homozygosity in each of the three *Metrosideros polymorpha* genetic clusters against both of the other genetic clusters.

Region ID	Genetic cluster with extended homozygous haplotypes	Pseudo-chromosome	Start position	End position	Length (bp)	Nr. 20-kb windows									Overlap with RAiSD outlier regions (kb)
						All	*D*_XY_ (G-I) outlier	*D*_XY_ (G-P) outlier	*D*_XY_ (I-P) outlier	*F*_ST_ (G-I) outlier	*F*_ST_ (G-P) outlier	*F*_ST_ (I-P) outlier	π outlier	Tajima’s *D* outlier	
G1	glaberrima	Mpol_Chr03	18,057,860	18,184,154	126,295	3	2**	1	NA	0	0	NA	0	0	0
G2	glaberrima	Mpol_Chr11	4,275,197	4,323,722	48,526	2	1	1	NA	0	0	NA	1	0	0
I1	incana	Mpol_Chr02	18,810,670	18,889,083	78,414	3	1	NA	1	1	NA	0	0	1	0
I2	incana	Mpol_Chr03	3,891,815	3,994,108	102,294	5	0	NA	0	0	NA	0	2*	1	0
I3	incana	Mpol_Chr06	2,206,825	2,324,704	117,880	6	2*	NA	1	0	NA	0	0	0	0
I4	incana	Mpol_Chr06	16,515,161	16,580,947	65,787	3	3**	NA	3**	0	NA	0	0	0	0
P1	polymorpha	Mpol_Chr01	20,313,337	20,489,061	175,725	8	NA	0	0	NA	0	0	7**	5**	124
P2	polymorpha	Mpol_Chr02	3,364,778	3,502,559	137,782	7	NA	0	0	NA	0	0	5**	3**	20
P3	polymorpha	Mpol_Chr06	24,179,678	24,248,660	68,983	3	NA	0	0	NA	0	0	1	2**	0

Overrepresentation of *D*_XY_, *F*_ST_, *π*, and Tajima’s *D* outlier windows in each extended haplotype homozygosity region examined with Fisher’s exact test

**: *p* < 0.01

*: *p* < 0.05.

Parenthesis of *D*_XY_ and *F*_ST_ indicate a pair of genetic clusters (G-I: the glaberrima and incana clusters; G-P: the glaberrima and polymorpha clusters; I-P: the incana and polymorpha clusters)

While a large *D*_XY_ with normal π suggests selection against gene flow from another genetic cluster, a small π with normal *D*_XY_ indicates a selective sweep in one genetic cluster e.g., [27–29]. We found signatures of selection against gene flow in the glaberrima and incana genetic clusters. The G1, I3, and I4 regions were significantly enriched with the top 5% *D*_XY_ windows calculated between the glaberrima and incana clusters (Table 1). The I4 region was also enriched with the top 5% *D*_XY_ windows between the incana and polymorpha clusters (Table 1). The G2 and I1 regions included *D*_XY_ and *F*_ST_ outlier windows, although these overlaps were not statistically significant (Table 1). The I4 region encompassed only two genes, *WUSCHEL-RELATED HOMEOBOX 4* (*WOX4*; Mpol2_06G0152700) and *NTL9* encoding a membrane-associated NAC transcription factor (Mpol2_06G0152800) (S2 Table).

In contrast, the signatures of selective sweep were found in one region in the incana cluster (I2) and three regions in the polymorpha clusters (P1–P3). The P1 and P2 regions significantly overlapped with the bottom 5% π windows and RAiSD outliers, and all the P1, P2, and P3 regions were among the lower 5% Tajima’s *D* windows in the polymorpha cluster (Table 1). Among the 22 genes in the P1 region, there were genes encoding relevant proteins to adaptation in the subalpine zone, such as *WRKY* transcription factor, Chlorophyll a/b binding protein, *JUNGBRUNNEN*, and Cytochrome b561 domain-containing protein.

Antagonistic pleiotropy, in which different alleles in the same genetic regions are selected in different populations, is another important process of adaptive evolution. In the three *F*_ST_ outlier windows found above, different clusters had different alleles at more than half SNPs embedded in a window. The most prominent signature was found in a window with large *F*_ST_ between the incana and polymorpha cluster, which included a gene putatively functioning in the RNA-directed DNA methylation pathway (Mpol2_07G0105400), membrane-associated kinase regulator (Mpol2_07G0105600), and one gene with unknown function (Mpol2_07G0105500) (S5 Fig).

### Demographic history

**Fig 5:**
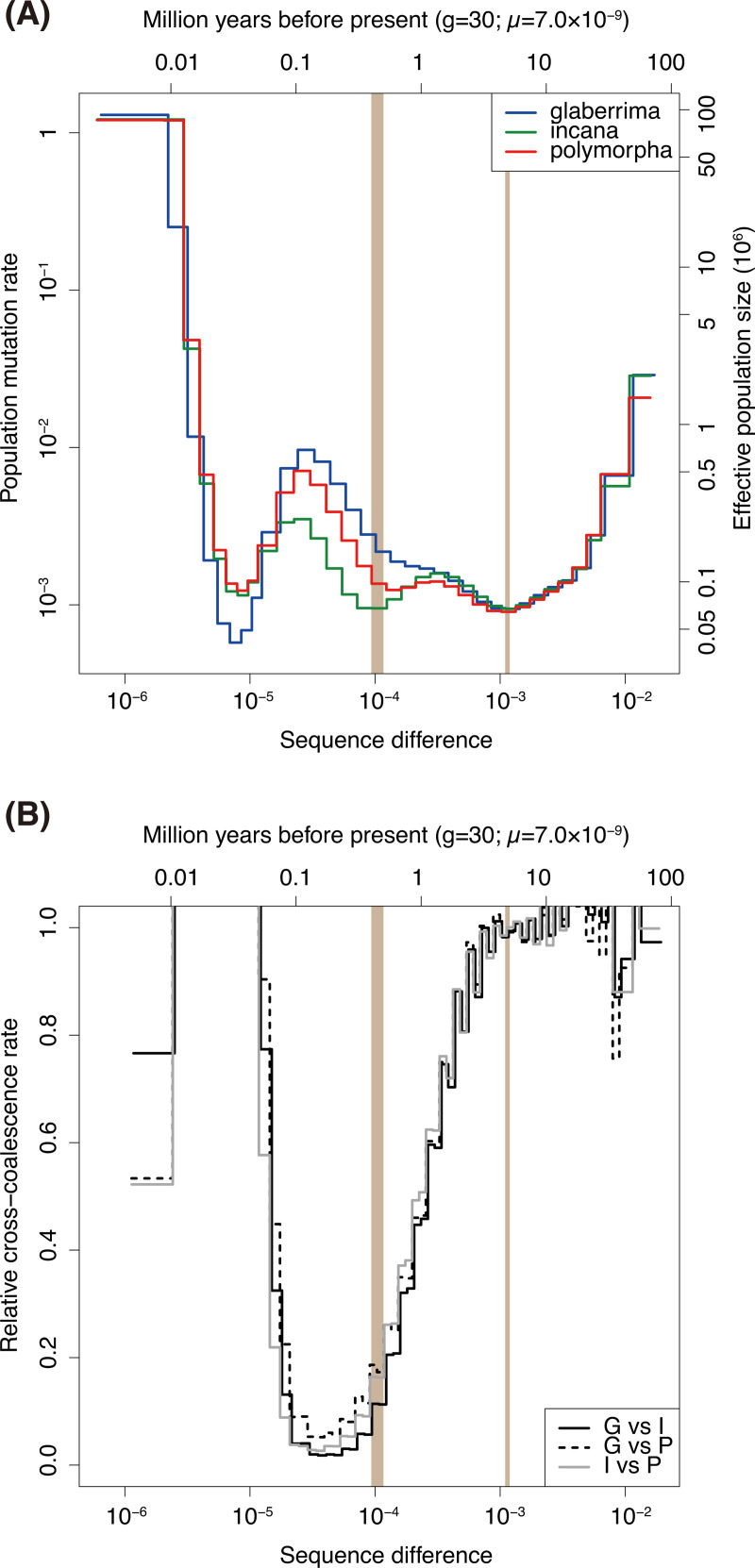
Multiple Sequentially Markovian Coalescent (MSMC)-based estimation of the past demography of the three *Metrosideros polymorpha* genetic clusters: the glaberrima cluster (G), incana cluster (I), and polymorpha cluster (P). **(A)** Historical change in the effective population size. **(B)** Relative cross calescent rate between genetic clusters. Two vertical bars (colored in beige) at 4.7–5.1 and 0.4–0.5 million years before present indicate the estimated time periods when the islands of Kauai and Hawaii emerged, respectively.

We conducted Multiple Sequentially Markovian Coalescent (MSMC) [30] analysis to estimate past effective population sizes and coalescent rates within and between the three clusters (Fig 5). During early generations around the sequence difference of 10^−3^, the effective population size (*N*_*e*_) of the three clusters corresponded very well and the cross-coalescent rates (CCRs) stabilized around one, strongly suggesting they were not separated populations (Fig 5A and 5B). The subsequent decreases in CCR indicated the genetic differentiation between the three clusters, although the orders were not clearly resolved (Fig 5B). The genetic differentiation between the clusters further proceeded and almost completed while the *N*_*e*_ increased gradually (Fig 5A and 5B). Then, a bottleneck, or a decrease in *N*_*e*_ occurred in all the three clusters. Notably, the CCR increased along with the bottleneck (Fig 5B), suggesting that gene flow increased between the clusters. Afterwards, the *N*_*e*_ of all three clusters increased and the CCR decreased again (Fig 5A and 5B). By using an estimated mutation rate of 7.0 × 10^−9^ per site per generation [31] and a generation time of 30 years following Izuno et al. [32], three genetic clusters diverged before the island of Hawaii emerged 0.4–0.5 million years ago (Mya) and the recent bottleneck occurred around 50,000 years ago.

**Fig 6:**
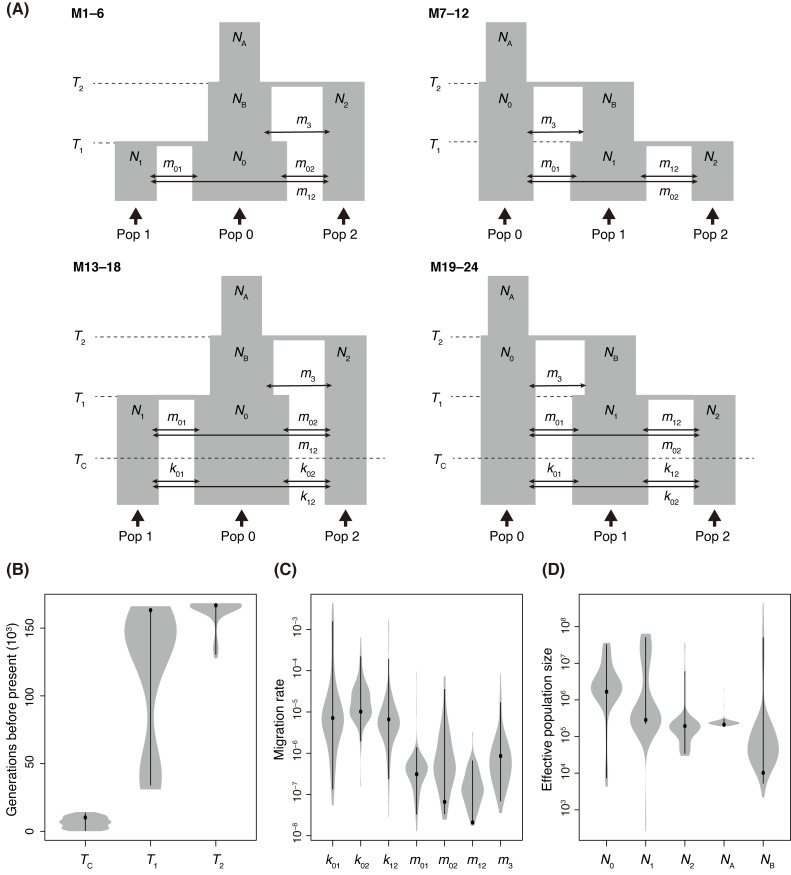
Site-frequency-based modeling of the population divergences in *Metrosideros polymorpha* on the island of Hawaii. **(A)** Schematic representation of simulated demographic models. Time points of divergence between three genetic clusters (*T*_1_ and *T*_2_), a time point when migration rates changed (*T*_C_), effective population sizes for present (*N*_0_, *N*_1_, and *N*_2_) and ancestral (*N*_A_ and *N*_B_) populations, and migration rates between genetic clusters (*k*_01_, *k*_02_, *k*_12_, *m*_01_, *m*_02_, *m*_12_, and *m*_3_) were inferred based on the multidimensional site-frequency spectrum observed in the glaberrima, incana, and polymorpha genetic clusters. The models M1–12 and M13–24 hypothesized the divergence after and before the colonization of the island of Hawaii, respectively. **(B–D)** Parameter estimates (black points) and ranges (gray shade) derived from the parametric bootstrap for the best model (M22). Black lines indicate 95% confidence intervals.

We further inferred the demography using site-frequency-based modeling [33]. The observed site-frequency spectrum was computed for each pair of the three genetic clusters (S6 Fig), and was fit to 24 possible models to estimate demographic parameters (Fig 6A). Models 1–12 hypothesized that all the genetic differentiation events occurred relatively recently and migration rate was constant after the split of the three genetic clusters. Models 13–24, on the other hand, hypothesized that the split between genetic clusters were followed by a change in migration rate, which was inspired by the MSMC analysis. As a result, M22 was the most likely scenario with the smallest Akaike’s information Criterion (AIC) (Table 2). In this model, the polymorpha cluster diverged from the glaberrima cluster 166,784 (95% confidence interval (CI): 130,580–168,219) generation ago, then the incana cluster diverged from the polymorpha cluster 163,216 (95% CI: 34,109–163,033) generations ago, although these split times were close each other and the CIs overlapped (Fig 6B). Assuming a mutation rate of 7.0 × 10^−9^ per site per generation [31] and 30 years per generation [32], these splits corresponds to 5.00 (95% CI: 3.92–5.05) million years ago (Mya) and 4.90 (95%CI: 1.02–4.89) Mya, respectively. At 10,713 (95% CI: 410–13,421) generations ago, which corresponds to 0.31 (95% CI: 0.01–0.40) Mya, the migration rates between the three genetic clusters increased about 23–314 fold (Fig 6C). This increase was equivalent to the change in population migration rate from 0.02–2.08 to 5.11–68.46 (S3 Table). A larger effective population size was estimated in the glaberrima cluster (*N*_0_) than the incana and polymorpha clusters (*N*_1_ and *N*_2_; Fig 6D), which is consistent with the largest genetic diversity and recent population expansion suggested by more negatively skewed Tajima’s *D* (Fig 3B and 3D). The difference between observed SFS and expected SFS under the best model deviated from 0, indicating even the best model misses some true demographic processes of the species (S6 Fig).

**Table 2:** Comparison of the 24 demographic models for the three *Metrosideros polymorpha* genetic clusters using Akaike’s information criterion (AIC).

Model	Pop0	Pop1	Pop2	Maximum ln(likelihood)	Number of parameters	AIC	Delta AIC
M1	G	I	P	-127172.5	11	585672.9	29217.3
M2	I	P	G	-127229.0	11	585933.3	29477.7
M3	P	G	I	-127286.5	11	586198.1	29742.5
M4	G	P	I	-127281.5	11	586174.7	29719.1
M5	I	G	P	-127261.8	11	586084.3	29628.7
M6	P	I	G	-127153.6	11	585585.7	29130.1
M7	G	I	P	-126330.1	11	581793.6	25338.0
M8	I	P	G	-127334.0	11	586416.5	29960.9
M9	P	G	I	-127219.8	11	585890.9	29435.3
M10	G	P	I	-127576.1	11	587531.8	31076.2
M11	I	G	P	-127356.7	11	586521.2	30065.6
M12	P	I	G	-127250.8	11	586033.5	29577.9
M13	G	I	P	-120975.1	15	557141.0	685.4
M14	I	P	G	-120873.1	15	556671.3	215.7
M15	P	G	I	-120944.8	15	557001.5	545.9
M16	G	P	I	-120967.7	15	557106.9	651.3
M17	I	G	P	-120983.8	15	557181.0	725.4
M18	P	I	G	-120858.9	15	556605.7	150.1
M19	G	I	P	-120890.9	15	556753.1	297.5
M20	I	P	G	-120992.7	15	557221.8	766.2
M21	P	G	I	-120972.1	15	557127.3	671.7
**M22**	**G**	**P**	**I**	**-120826.3**	**15**	**556455.6**	**0.0**
M23	I	G	P	-121018.4	15	557340.6	885.0
M24	P	I	G	-121035.5	15	557418.9	963.2

G, I, and P indicates the glaberrima, incana, and polymorpha cluster, respectively.

## Discussion

### Core evolutionary units comprising the *M*. *polymorpha* forests on the island of Hawaii

Our extensive genome sequencing revealed three core genetic clusters comprising the continuous *M*. *polymorpha* forests on the island of Hawaii adapted to different environments: the glaberrima cluster in the wet cloud forest, the incana cluster in the dry and early successional forests, and the polymorpha cluster in the subalpine zone (Fig 2). Each of the major genetic clusters harbored substantial genetic and phenotypic diversity (Figs 1 and 3). In the glaberrima and incana cluster, a further genetic differentiation was observed, possibly due to geographic isolations within clusters (S1 Fig). The glaberrima cluster accommodated riparian var. *newelii* (Fig 2). Several studies [16,18,20] analyzed a substantial number of var. *newelli* samples and reported evident differentiation in the allele frequency between var. *glaberrima* and var. *newelii*. The distinctive morphology of var. *newelii* is the consequence of local adaptation to riparian environments [23]. Our results indicated that the genetic differentiation was smaller than that among the three major genetic clusters and supported that the adaptation to riparian environments emerged from var. *glaberrima* [16]. In the polymorpha cluster, glabrous var. *nuda* shared neutral genetic variations with pubescent var. *polymorpha* (Figs 2 and S1). Therefore, differences in leaf pubescence and physiological characteristics [34] could be attributed to a limited number of genetic differences. Further studies are required to determine the ecological and genetic factors underlying the scarcity of var. *nuda* plants in the subalpine zone.

### Natural selection associated with ecological divergence

By combining the results from different methodologies for selection scan, we identified two to four EHH regions in each genetic cluster that showed a signature of selection against gene flow or selective sweep (Table 1). These regions encompassed several candidate genes responsible for adaptation in each cluster (S2 Table). For example, the I4 region, which showed a signature of selection against gene flow between the other two genetic clusters, included two genes (Tables 1 and S2): *WOX4* and *NTL9*. *WOX4* is involved in various developmental processes, including an early stage of leaf development in rice [35], the maintenance of vascular stem cells in *Arabidopsis* [36], and secondary growth through regulating cambial cell division in *Populus* [37]. *NTL9* plays a role in plant immunity [38] and regulates osmotic stress signaling during leaf senescence [39]. One or both of these genes must be important for the incana cluster to colonize young substrates as a pioneer plant shortly after volcanic eruptions.

Other candidate genes were found in the P1 region, which showed a signature of selective sweep in the polymorpha cluster (Table 1). Because the polymorha cluster is exposed to strong radiation without clouds (Fig 2D), the regulation of light and oxidative stress are likely to be important. Chlorophyll a/b binding protein (*LCHB*; Mpol2_01G0166500), which harvests sunlight [40,41], and Cytochrome b561 domain-containing protein (Cyt-b561; Mpol2_01G0167500), which prevents damage from excess light under drought conditions [41], are good candidate genes for controlling light utilization. In addition to these two genes, *WRKY28* (Mpol2_01G0166100) is likely to respond to oxidative stress as its expression is increased under oxidative stress in *A*. *thaliana* [42–47]. In the subalpine zone, there is a deficiency of soil nitrogen under cool temperatures [8,48], making nitrogen management another significant consideration for the polymorpha cluster. Transcription factor *JUNGBRUNNEN1* (Mpol2_01G0168500), whose overexpression delays leaf senescence in *A*. *thaliana* [49], could be related to longer leaf longevity in the polymorpha cluster compared with other genetic clusters: the leaf longevity of the polymorpha cluster and the other two genetic clusters was estimated to be ca. 8.5 and 2.2 years, respectively [12]. This co-localization of genes with similar selective forces could have contributed to accelerate adaptation [50,51].

The nine EHH regions (Table 1) could be among the most significant genomic regions for the adaptive evolution of the species. Besides these, substantial genomic regions showed the signals for EHH, genetic differentiation, and selective sweep in each analysis, but rarely overlapped with those found in different analyses (Figs 4, S3 and S4). This indicated the extent and time scales of selection varies across the genomes, as the different time scales of selection are measured by different parameters [52]. Genomic regions subject to recent selection could show the signatures of selective sweep but may not be differentiated between genetic clusters. Likewise, old haplotypes relevant to adaptation in the past could still be genetically differentiated from other genetic clusters but the homozygosity may not be extended as long as before.

There were a few signatures of antagonistic pleiotropy among *F*_ST_ outliers (S5 Fig). In the most of *F*_ST_ outliers, alleles could show fitness advantage in one genetic cluster (i.e., conditional neutrality; [53]) or allelic trade-offs could occur in narrow genomic regions within a 20-kb window. The genes around the genomic region showing allelic trade-offs has broad or unknown functions (S5 Fig), therefore, the role of antagonistic pleiotropy in the evolution of *M*. *polymorpha* was unclear.

### Divergence and admixture history

How *M*. *polymorpha* adapted to the diverse environments on the Hawaiian Islands is a long-standing question. A scenario in which multiple, ecologically-diverged *M*. *polymorpha* lineages independently colonized each island was suggested because of similar ecological and morphological adaptations on different islands. However, genetic differentiation between varieties within islands was negligible compared to that between islands [5,20], suggesting that the ecological divergence in *M*. *polymorpha* occurred on each island. Our data suggest a new scenario in which these two scenarios may not be mutually exclusive.

Previous phylogenic studies suggested that all the Hawaiian *Metrosideros* species are monophyletic and most closely related to a species from southern Polynesia [5,6]. Thus, it is possible that a reduction in *N*_e_ found around 5 Mya was associated with the introduction into the Hawaiian Archipelago through long-distance dispersal. Considering that long-distance dispersal does not always result in a temporal reduction in *N*_e_ [54,55], the ecological windows open to the ancestral populations of the species was likely to be small. The immigration could rely on a rare dispersal vector, such as jet stream [56,57], and/or only a fraction of immigrants survived on new environments [58].

After the bottleneck, the three genetic clusters split and the connectivity was progressively lost between clusters (Fig 5B). A major question remains as to whether the split occurred within the island or in advance in different islands. By assuming a generation time of 30 years and a mutation rate estimated for *Arabidopsis*, the split was estimated to start 4.9–5.1 Mya in fastsimcoal (Fig 6B) and a few to five Mya in MSMC (Fig 5B). Therefore, this split most likely dates before the emergence of the island of Hawaii about 0.4–0.5 million years ago. Care should be taken in interpreting the results because it is still possible that some clusters diverged on the island of Hawaii when considering different generation times and mutation rates (S7 Fig). Very close point estimations of *T*_1_ and *T*_2_ in fastsimcoal modelling (Fig 6B) and mostly coincident CCR traces around 1–5 Mya (Fig 5B) suggested that the divergence between three genetic clusters likely occurred in a short period of time and/or that the current data are insufficient to resolve the orders of divergence.

Among the three clusters, the archipelago-wide distribution of var. *glaberrima* could be closely related to the ancestral form that migrated to the archipelago, which is consistent with the best fitted model (M22; Fig 6). Var. *glaberrima* grows in wet and late-successional forests and the sort of habitat appears on every island even after the islands subside and become eroded. The central position (around 100–1500 m above sea level) in the elevational range of the species distribution could lead to high genetic diversity through increased hybridization between other genetic clusters or varieties. As a previous implication based on microsatellite polymorphism [20], this large, genetically rich, ancestral genetic cluster could have served as the genetic source of adaptive diversification in the species. Currently, the polymorpha cluster (i.e., var. *polymorpha* and var. *nuda*) is not found on Kauai Island, and it is rare on Oahu Island [17], where there is no remaining subalpine zone. Likewise, the incana cluster (i.e., var. *incana*) does not exist on Kauai Island and remains in small populations on Oahu Island [17], where forest succession proceeded and suitable habitat disappeared. However, as all Hawaiian Islands emerged through volcanic eruption and likely subsided 2,000–4,000 m compared to their historical maximum height [59], it is possible that the ancient bare lava flows or subalpine environments over the inversion layers provided opportunities for ecological differentiation of the incana and polymorpha clusters in the past.

Island organisms that experienced a population bottleneck during initial colonization often reduce the *N*_e_ [60]. Relatively small area of islands tends to reduce the organisms’ dispersal ability [61]. *Metrosideros polymorpha*, however, remarkably increased the *N*_e_ since the first immigration to the archipelago (Figs 5A and 6D) and still shows high dispersal ability [62]. A series of volcano eruption in the Hawaiian Islands could make it possible for the species to successively expand the distribution and maintain the genetic variation. The ecological divergence could be a consequence of the large *N*_e_ through history, in which natural selection acts effectively [63].

Our fastsimcoal modelling and MSMC analysis both suggested an increase in genetic connectivity between clusters in a relatively recent time, estimated during 0.01–0.4 Mya (Figs 5 and 6). This indicated once differentiated genetic clusters experienced a secondary contact. Importantly, the secondary contact occurred in association with a bottleneck (Fig 5). The weak genetic isolation between the *M*. *polymorpha* clusters is consistent with the lack of strong reproductive isolation in current populations, which is indicated by the absence of geographic barriers, especially on young islands [64], the mostly shared pollinators and flowering period [62,19], and the slightly reduced hybrid fertility [19]. Assuming weak reproductive isolation in the past, the low CCR during older periods could suggest that the clusters were in allopatry on single or different islands. The timing of the bottleneck and loss of isolation possibly coincide with the last glacial maximum (LGM; Fig 5). During the LGM, the climate of the Hawaiian Islands was cooler and drier than today: the sea surface temperature was 2°C cooler [65] and the treeline on Mauna Kea was depressed to an altitude of 2,000 m because of the depressed inversion layer level [66]. Arid conditions may have spread around the island of Hawaii, given that xerophytic plants dominated the vegetation of the island [67]. Consistent with the reduced effective population sizes of the MSMC-based estimation (Fig 5), less *Metrosideros* pollen was detected in the LGM compared to that during more recent time periods, even at an altitude of 465 m on the Oahu shoreline [68]. The decrease in the suitable habitat of the species as a whole might have allowed a parapatric distribution and promoted secondary contact among the three genetic clusters. The effective population sizes of all three *M*. *polymorpha* genetic clusters increased remarkably since the bottleneck (Fig 5), although it is possible that the values in the recent past were overestimated and unreliable due to the limited sample sizes used for MSMC and/or phasing errors [69–71]. In the post-glacial period, the temperature became warmer and precipitation increased due to the inversion level rising [72]. Given the suggested dominance of rainforest vegetation on the island based on the pollen record [67], suitable areas for the glaberrima cluster likely increased. The eruptions of Mauna Loa and Kilauea [21] and a fluctuating inversion layer level in more recent years [72] may have provided more opportunities for the incana or polymorpha clusters to increase their population sizes.

The differentiation of the three clusters before the emergence of the island of Hawaii does not necessarily contradict the genome-wide similarity of plants on each island. Discrepancy between population history and genealogy of adaptive genes was also reported in recent genomic studies [73,74]. In *M*. *polymorpha*, we hypothesize that the three genetic clusters diverged through environmental adaptation and then came into close contact on the island of Hawaii. While the genomic regions responsible for local adaptation remained differentiated, the majority of the genome may have become undifferentiated due to weak reproductive isolation. The candidate regions under selection, as described above, provide the key to examine the hypothesis of genetic divergence through environmental adaptation when integrated with the sequence data from different islands.

## Methods

### Plant materials and trait measurement

In 2016–2017, 66 samples were collected from 51 sites that covered almost all the *M*. *polymorpha* populations on the island of Hawaii. Leaf functional traits, including leaf area, leaf tissue mass per area, and trichome mass per area, were quantitatively measured following Tsujii et al. [12]. Each sample was classified into five known varieties following Dawson and Stemmermann [17]: var. *glaberrima*, var. *incana*, var. *polymorpha*, var. *nuda*, and var. *newelii*. Plant samples were collected under the research permissions from U.S. National Park Service, Division of forestry and Wildlife in State of Hawaii, and Kohala Ranch. We also included four samples from Stacy et al. [18] to compensate for the habitat range of *M*. *polymorpha* on the island. The four samples showed typical morphological characteristics of varieties, but qualitative measurements of the leaf traits were not taken.

### Whole-genome sequencing

Genomic DNA was extracted using NucleoSpin Plant II (Macherey-Nagel, Düren, Germany). After the genomic DNA was fragmented into approximately 350 bp fragments using a sonicator (E220 or LE220, Covaris, Woburn, MA, USA), sequencing libraries were synthesized using NEBNext Ultra II DNA Library Prep Kit for Illumina (New England BioLabs, Ipswich, MA, USA) or a TruSeq DNA PCR-Free High Throughput Library Prep Kit (Illumina, San Diego, CA, USA) following the manufacturer’s protocols. Each DNA library was tagged with distinctive barcode sequences and equimolarly pooled into a tube. Of the 70 samples, 30 were sequenced in four lanes in HiSeq2500 (Illumina) with the paired-end 125 bp chemistry, while the remaining were sequenced in four lanes in HiSeq X Ten (Illumina) with the paired-end 150 bp chemistry. Raw sequence data are deposited in the DNA Data Bank of Japan (accession: DRA011715). We obtained an average of 10.3 G bases (range: 2.95–34.4 Gb) per sample.

### Quality control, mapping, and variant calling

After trimming adapter sequences and low-quality bases using Trimmomatic (ver. 0.33) [75], the clean reads were aligned against the *M*. *polymorpha* reference genome sequence [22] using the BWA-MEM algorithm (ver. 0.7.12-r1039) [76]. For two samples (HI_26 and HI_28 in S1 Table) with more than 70 million reads, 60 million clean read pairs were randomly selected and used for mapping due to limited computational capacity. Approximately 98% of the raw data passed quality filtering and 95% of the data aligned with the reference genome sequence. PCR-derived duplicated reads in the alignments were marked using Picard (ver. 2.6; http://broadinstitute.github.io/picard/). We obtained alignments with an average of 29.6-fold (range: 9.5–52.5 fold) coverage.

Nucleotide polymorphisms against the reference sequence were called for each sample using GATK HplotypeCaller (ver. 3.6) [77]. The resulting variant call format files were used to identify variant and invariant sites among the 70 samples. We ignored sites on the repeat sequences and transposable elements and retained sites satisfying specific qualities: QD >2.0, MQ >30.0, MQRankSum >−12.5, FS <60.0, SOR <3.0, and ReadPosRankSum >−8.0. We regarded genotypes as missing if the depth per genotype was extremely low (<5) or high (>100). For variant sites, SNPs with more than three alleles were excluded from the dataset and the effect of SNPs on gene functions were estimated using SnpEff (ver. 4.3T) [78] based on the *M*. *polymorpha* gene annotation [22]. A total of 243,376,950 sites, including 231,298,492 monomorphic sites and 12,078,458 bi-allelic SNPs, were used for subsequent analyses.

### Population structure

The population structure among the 70 *M*. *polymorpha* plants was assessed using ADMIXTURE (ver. 1.3) [24], principal component analysis (PCA), and redundancy analysis (RDA). In the ADMIXTURE analysis, the 70 plants were clustered assuming 1–10 ancestries (*K* = 1–10) with 10 replicated runs per *K*. We used 57,196 four-fold degenerate SNPs, in which the minor allele frequency was larger than 0.05, LD between any sites within 50 bp did not exceed 0.5, and less than 20% missing data was allowed per SNP. In the PCA, we used 16,972 four-fold degenerate SNPs, which were retained after removing SNPs with missing genotypes from the SNP dataset used in ADMIXTURE.

In order to evaluate the effect of geographic and environmental distances on the genetic differentiation, we modeled the genetic variation among the 70 plants with geographic (GEO) and environmental (ENV) distance matrices and conducted RDA to partition the variation. Genetic variation was represented by the genotypes at the same SNPs used for PCA. The environmental distances were estimated from the scaled 12 environment variables, including substrate age, mean annual air temperature, annual precipitation, cloud frequency, net radiation, albedo, Penman-Monteith potential evapotranspiration, soil evaporation, transpiration, vegetation height, and vapor pressure deficit. These data were obtained from the Geography Department of the University of Hawaii (http://climate.geography.hawaii.edu) [79] and the United States Geological Survey (https://pubs.usgs.gov/of/2007/1089/) [21]. The geographic distances were represented by 13 distance-based Moran’s eigenvector map (dbMEM), which is obtained from GPS coordinates using the *dbmem* function in *adespatial* package (ver. 0.3.14) [80]. In order to avoid overfitting in the subsequent RDA, forward selection was performed for each of environmental and geographic variable sets using the *ordiR2step* function in *vegan* package (ver. 2.5.7) [81]. In the end, six environmental variables, including mean annual air temperature (Temp in Fig 2D), soil evaporation (Soil evapo), annual precipitation (Rainfall), substrate age (Lava age), cloud frequency (Cloud frq), vapor pressure deficit (VPD), and six dbMEMs were used for ENV and GEO matrix, respectively, in the following RDA. Proportion of genetic variation explained by the GEO and ENV matrices were estimated using *varpart* function in *vegan* package based on the full and partial RDA models. Statistical significance of explanatory variables in each model was examined by a permutation test (999 permutations).

### Genetic diversity

In the subsequent analyses, we focused on 40 samples with >90% ancestry in the population clustering, assuming three ancestries (14, 14, and 12 samples for the glaberrima, incana, and polymorpha clusters, respectively; S1 Table). Bi-allelic SNPs with <20% missing data in the 40 samples were used for calculation.

LD decay was estimated using PopLDdecay [82], in which we further removed SNPs with a minor allele frequency of less than 0.1 and calculated the squared correlation coefficient (*r*^2^) between SNP genotypes at a maximum of 100 kb apart. The ROH, i.e., the stretches of homozygous genotypes, and Tajima’s *D* in 20-kb nonoverlapping sliding windows were calculated for each genetic cluster using VCFtools [83]. Nucleotide diversity within each genetic clusters (*π*) in 20-kb sliding windows using pixy [84].

### Genome scan for selection

Genomic regions with a signature of selection were searched based on haplotype homozygosity estimates, sweep statistics, local deviation of *F*_ST_, and diversity and divergence parameters such as *π*, Tajima’s *D*, and *D*_XY_.

If the frequency of a beneficial allele increases rapidly, the haplotype homozygosity around the allele becomes more extended than expected with normal recombination. Therefore, significantly different haplotype homozygosity between genetic clusters indicates positive selection affecting one genetic cluster. For each pair of three *M*. *polymorpha* genetic clusters, we calculated cross-population extended haplotype homozygosity (xp-EHH) around SNPs with more than four minor alleles in each pair using the R package *rehh* (ver. 3.1.0) [85]. As this analysis requires haplotype information, haplotypes of individuals in each genetic cluster were statistically inferred from genotype data using WhatsHap (ver. 0.18) [86] and Shapeit (ver. 4.1.2) [87]. SNPs were recognized as outliers if the p-value of xp-EHH score between each of genetic cluster pairs was less than 10^−6^. Around each outlier SNP, genomic regions showing EHH in one genetic cluster against the other was identified using *calc_furcation* function in *rehh* [85] and consecutive EHH regions were merged. The glaberrima cluster showed EHH at 13 and 23 regions against the incana and polymorpha cluster, respectively. Likewise, the incana cluster showed EHH at 30 and 21 regions against the glaberrima and polymorpha cluster, respectively, and the polymorpha cluster showed EHH at 28 and 13 regions against the glaberrima and incana cluster, respectively. The subsequent analyses focused on EHH regions that were found in comparison with both of other genetic clusters, because these EHH regions can encompass genomic signatures of cluster-specific adaptation.

The signatures of selective sweep were searched using RAiSD (ver. 2.0) [26]. RAiSD calculates a Mu statistic, which is an integrated score dependent on the extent of polymorphism, LD, and the change in SFS, for a stretch of sequences including 50 SNPs. Higher Mu statistic values indicate the genetic regions are more likely to be affected by selective sweep. RAiSD was carried out for each genetic cluster. Only SNPs without missing data were recognized as polymorphisms and all the remaining sites were treated as monomorphic sites. To avoid high Mu statistics calculated for windows with considerable missing data, we excluded RAiSD-defined sequence windows that overlapped with long (>1 kb in length) no-record regions or included short (100–1000 bp) no-record regions more than 20% of the total window length. After the filtering, 4,603,611, 2,495,674, and 2,195,041 windows were retained for the glaberrima, incana and polymorpha cluster, respectively. Mu statistics more than 20 standard deviation from the mean was defined as significant outliers.

Local deviation of genetic differentiation from genome-wide one was evaluated by *F*_ST_ outlier scan. We characterized each 20-kb window with an *F*_ST_ vector, which is composed of three pairwise *F*_ST_ values and embedded within a three-dimensional numerical space. The local deviation of each window was measured based on *k*-nearest neighbor (kNN) method [88]. To exclude the bias of low frequent alleles, SNPs with less than four minor allele counts in each cluster pair were removed for calculation (3,090,901, 2,912,614, and 2,843,930 SNPs were used to calculate *F*_ST_ between the gaberrima and incana, glaberrima and polymorpha, and inacna and polymorpha clusters, respectively) and windows with less than 20 such SNPs were removed. Pairwise *F*_ST_ [89] were calculated using VCFtools and negative or missing values were converted into zero. With a weighted kNN method [90], the degree of local deviation (hereafter, kNN outlier score) is measured for each window by the sum of dissimilarity between the other nearest *k* windows, where *k* = 6993 was determined based on the diagnostic plot provided by Pfeifer et al. [88]. Windows within the top 0.5% kNN outlier scores and more than 0.5 of delta *F*_ST_, which is the difference between an *F*_ST_ vector and the medoid of the all pairwise *F*_ST_ vectors [88], were recognized as outliers.

We further computed nucleotide diversity within clusters (*π*), Tajima’s *D*, and the absolute genetic differentiation between clusters (*D*_XY_) in 20-kb sliding windows using pixy [84] and VCFtools [83]. Windows within the bottom or top 5% of the whole-genome distribution were recognized as outlier regions (bottom 5% for *π* and Tajima’s *D*; top 5% for *D*_XY_).

The results from the four approaches were combined and EHH regions with at least one significant evidence from other methodologies were further discussed. We examined whether each EHH region was enriched with outlier windows of *D*_XY_, *F*_ST_, *π*, and Tajima’s *D* using Fisher’s exact test.

To find genomic signatures of antagonistic pleiotropy, we screened *F*_ST_ outlier windows. Windows in which allele frequency differed > 0.95 in a cluster pair at more than half of SNPs were recognized as candidate regions with signatures of antagonistic pleiotropy.

### Demographic history

The historical change in the effective population size and gene flow was first inferred using a model-free approach. The coalescent times of haplotypes within and across genetic clusters were computed using MSMC (ver. 2.1.1) [30] with eight and four haplotypes, respectively, from each genetic cluster. Output estimates were converted to their real size and time with an assumed generation time of 30 years and a mutation rate of 7.0 × 10^−9^ per site per generation. The generation time of 30 years could be a conservative estimate, as *M*. *polymorpha* is a pioneer species colonizing early successional substrates and a previous study assumed the generation time to be 10–20 years [16]. It is reported that the mutation rate per generation in woody species could be similar or slower compared with annual plant species [91], while a faster mutation rate of 3.75 × 10^−8^ per site per generation is assumed for *Populus* [92]. These uncertainties in generation time and mutation rate were considered following Sakojärvi et al. [93]. Briefly, after computing a range of mutation rate per site per year of *A*. *thaliana* based on relevant reports [31,94–96], the values were divided by six as Tuskan et al. [97], and then multiplied by the plausible generation times of *M*. *polymorpha* (10 and 30 years). This calculation led a range of mutation rate of 2.9–9.5 × 10^−9^ and 8.8–28.5 × 10^−9^ for a generation time of 10 and 30 years, respectively.

We then hypothesized 24 demographic models to estimate demographic parameters using SFS (Fig 6A). The parameters included effective population sizes (*N*_0_, *N*_1_, *N*_2_, *N*_A_, and *N*_B_), time points of population divergence (*T*_1_ and *T*_2_), a time point when migration rates changed (*T*_C_) and migration rates between genetic clusters (*k*_01_, *k*_02_, *k*_12_, *m*_01_, *m*_02_, *m*_12_, and *m*_3_). The models M1–12 hypothesized that the divergence among the three genetic clusters occurred relatively recently, thus the search range for *T*_2_ was set to be less than 15,000 generations before present (450,000 years with an assumed generation time of 30 years). This corresponds to the scenario that the three genetic clusters diverged after the colonization of the island of Hawaii. On the other hand, the models M13–24 hypothesized that the divergence had occurred before the colonization of the island and the migration rates between genetic clusters changed after that, thus the search range for *T*_2_ and *T*_C_ was set as >15,000 and <15,000 generations, respectively. We simplified the models by assuming constant effective population sizes and unidirectional migration rates to reduce computational effort. Using fastsimcoal2 (ver. 2.6) [33], the parameters were estimated based on the 3D-site-frequency spectrum (SFS) of the minor allele frequency observed in the three genetic clusters (S6 Fig). The SFS measures were calculated using a random set of 500,000 intergenic sites that were located >10 kb from exons and did not include missing data in the 40 samples. The 500,000 sites included 35,656 bi-allelic SNPs (7.10%). In each model, 40 independent runs of 100,000 coalescent simulations, with a maximum of 40 cycles of the likelihood maximization, were conducted. The most likely scenario was determined based on the AIC. The observed and expected SFSs under the best model were compared using a script provided in ∂a∂i [98]. For the best fit model, the 95% confidence interval of parameters was estimated with the 100 parametrically bootstrapped SFSs. The mutation rate was set as 7.0 × 10^−9^ per site per generation [31].

## Supporting information

S1 Table Summary of 70 *Metrosideros polymorpha* samples in this study. Sample identifications, localities (including GPS coordination), leaf trait data, and variety classification are shown.
(XLSX)

S2 Table List of genes overlapping with the candidate genomic regions under selection (G1–G2, I1–I4, and P1–P3). (XLSX)

S3 Table Population migration rates based on migration rates and effective population sizes estimated in the coalescent modeling. (XLSX)

S1 Fig Population clustering inferred with ADMIXTURE assuming 2–6 ancestries. G, I, P, NW, ND, and Un indicate var. *glabrrrima*, var. *incana*, var. *polymorpha*, var. *newelii*, var. *nuda*, and unclassified respectively. Letters in parenthesis indicate volcanos where samples were collected (KL: Kilauea, ML: Mauna Loa, MK: Mauna Kea, HL: Hualalai, KH: Kohala). Samples used for the coalescent simulations and selection scans are indicated with an asterisk.
(EPS)

S2 Fig Cross-validation errors in the ADMIXTURE analyses with *K* = 1–10. (EPS)

S3 Fig Manhattan plot of Mu statistics calculated in RAiSD. Horizontal dashed lines indicate the thresholds for outlier SNPs (Mean + 20 SD).
(TIFF)

S4 Fig **Manhattan plot of *F***_**ST**_ **between (A) the glaberrima and incana clusters, (B) the glaberrima and polymorpha clusters, and (C) the incana and polymorpha clusters.** Significantly deviated *F*_ST_ from the genome-wide distributions were indicated in red.
(TIF)

S5 Fig Genetic diversity and differentiation parameters around a candidate region for antagonistic pleiotropy found between the incana and polymorpha cluster. Allele frequency of non-reference allele (frq(ALT)), *π*, Tajima’s *D*, *F*_ST_, and *D*_XY_ around the three genes overlapped with an outlier 20-kb window were shown.
(EPS)

S6 Fig Observed and expected site-frequency spectrum (SFS) under the best demographic model of three *Metrosideros polymopha* genetic clusters on the island of Hawaii. (A) Observed and (B) expected two dimensional SFSs for each pair of the three genetic clusters. (C) Residuals between the expected and the observed SFS. (D) Histograms of residuals.
(EPS)

S7 Fig MSMC plots with different generation times and mutation rates. (EPS)
