# Supplementary figures and images for: Demography and selection analysis of the incipient adaptive radiation of a Hawaiian woody species
Source: PLoS Genet. 2022 Jan 21;18(1):e1009987. doi: 10.1371/journal.pgen.1009987 (PMC8782371; doi:10.1371/journal.pgen.1009987)

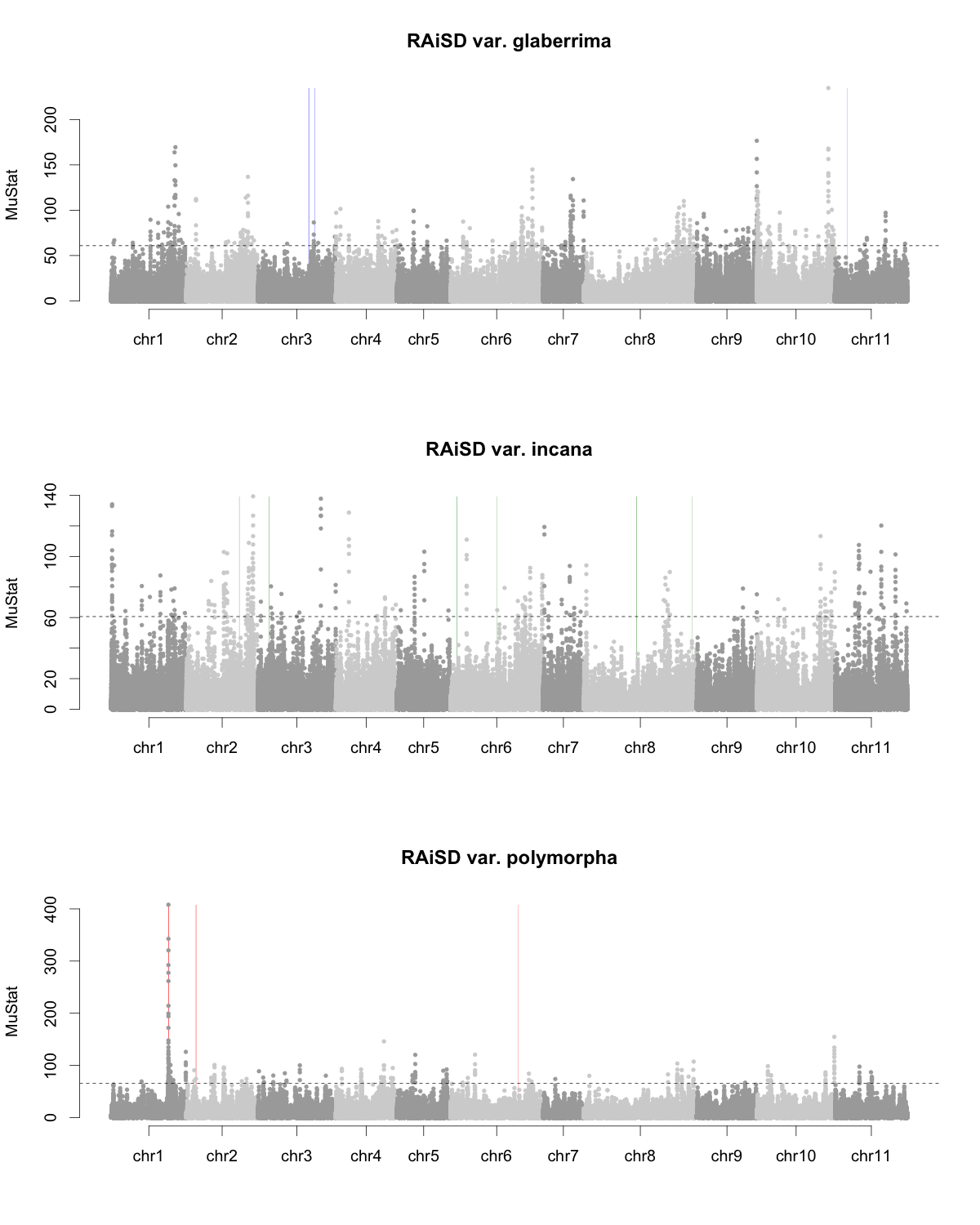

Supplement: S3 Fig — Horizontal dashed lines indicate the thresholds for outlier SNPs (Mean + 20 SD). (TIFF) [file pgen.1009987.s006.tiff]

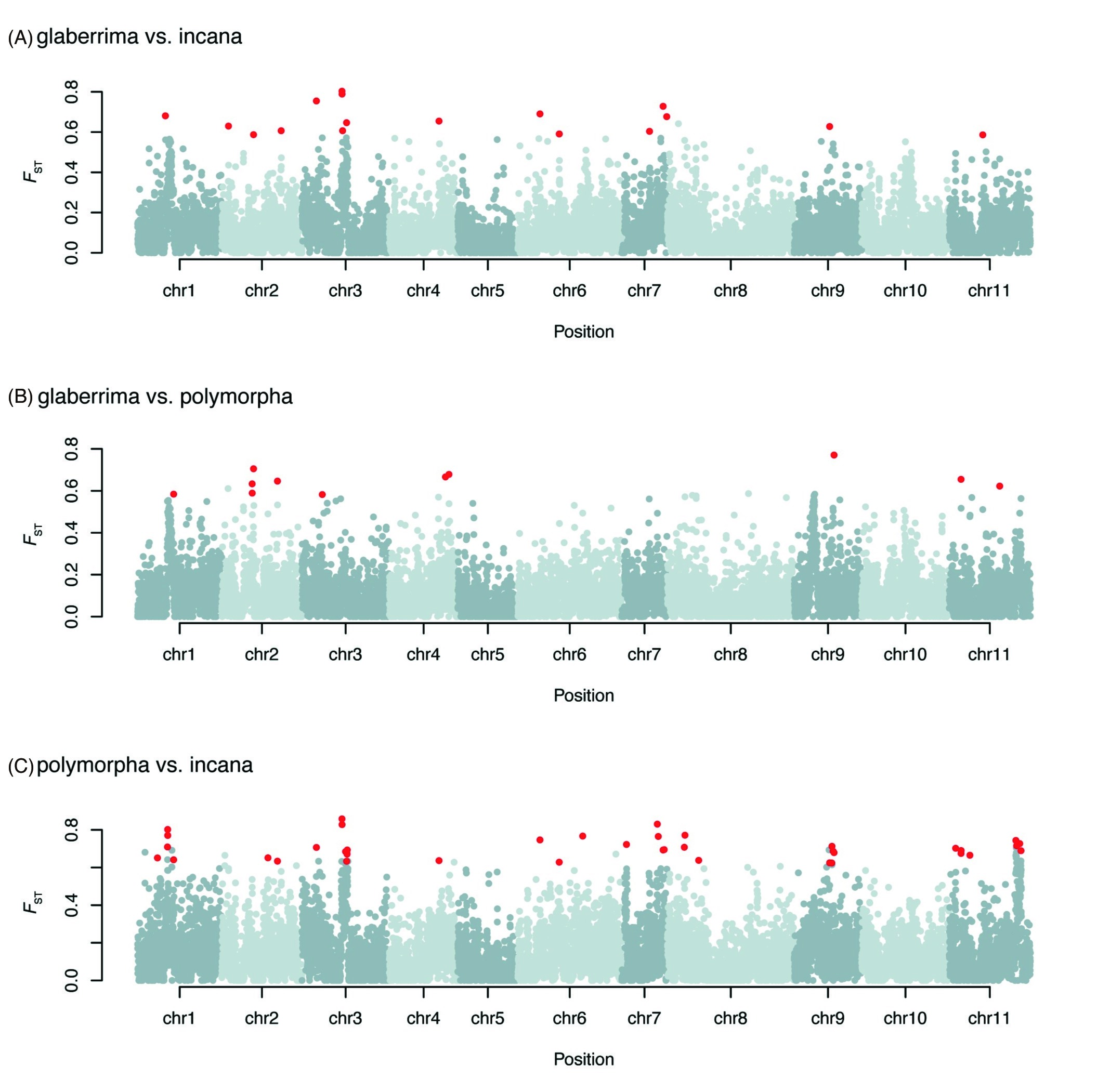

Supplement: S4 Fig — Manhattan plot of FST between (A) the glaberrima and incana clusters, (B) the glaberrima and polymorpha clusters, and (C) the incana and polymorpha clusters. Significantly deviated FST from the genome-wide distributions were indicated in red. (TIF) [file pgen.1009987.s007.tif]
